# Revision arthroscopic surgery after rotator cuff repair with a collagen graft: histologic evaluation of biopsy specimens from two patients

**DOI:** 10.1016/j.xrrt.2022.02.008

**Published:** 2022-03-31

**Authors:** Larissa E. Wietlisbach, Adnan N. Cheema, Jui-Han Huang, Xunda Luo, G. Russell Huffman

**Affiliations:** a Perelman School of Medicine at the University of Pennsylvania, Philadelphia, PA, USA; b Division of Shoulder and Elbow Surgery, Department of Orthopaedic Surgery, Mayo Clinic, Rochester, MN, USA; c Department of Pathology and Laboratory Medicine, Hospital of the University of Pennsylvania, Philadelphia, PA, USA; d Department of Orthopaedic Surgery, Hospital of the University of Pennsylvania, Philadelphia, PA, USA

**Keywords:** Rotator cuff, Collagen, scaffold, Tendinopathy, Inflammation, Bursitis, Integration

Rotator cuff tendinopathies are common and can be a significant source of pain and loss of shoulder function.^6^^,^^7^^,^^14^ Particularly, symptomatic advanced rotator cuff tendinopathy in the absence of a full-thickness tear presents a therapeutic challenge, as surgical options include débridement procedures that do not necessarily address the tendinopathy itself. A similar treatment pitfall applies to partial rotator cuff tears in that rotator cuff tear completion paired with repair opens the possibility of creating a full-thickness defect that does not heal as a complication. One alternative is a repair of the partial tear without completion, although practices vary due to the lack of clear evidence for one optimal treatment recommendation.^8^ New therapeutic modalities that offer biological enhancement of diseased tendons have emerged and show promise in the treatment of advanced rotator cuff tendinopathy.^10^ One such modality is an absorbable collagen implant. These implants may be used for tendon augmentation in the setting of repairs of full-thickness rotator cuff tears, as well as to improve the biological milieu in the surgical treatment of intra-tendinous tendinopathy.^1^^,^^10^^,^^11^^,^^13^^,^^15^ These implants have demonstrated improved surgical outcomes such as decreased pain and loss of function and lower revision/re-operation rates.^4^^,^^9^^,^^13^

The collagen graft comprises type 1 collagen and serves as a scaffold that is thought to structurally support the repair, thereby decreasing the tensile forces across new and fragile tissue at a repair site, in conjunction with inducing tendon tissue growth.^11^^,^^14^ In a sheep study, the graft’s porosity has been shown to promote host tissue ingrowth into the graft, forming mature tendon-like tissue and thickening the rotator cuff tendons.^15^ Similarly, histologic evaluation of seven patients’ repair augmented with a collagen graft showed findings of host tissue ingrowth within the graft and mature tissue formation and organization along the graft.^1^ The new and thicker tissue is believed to offload further and decrease rotator cuff strain, optimize healing potential, and reduce the rate of tendon re-tear.^1^^,^^15^

Multiple clinical studies have demonstrated that tendon repair augmented with a collagen implant induces significant tendon thickening and tear resolution on MRI by 12 months follow-up.^2^, ^3^, ^4^, ^5^^,^^12^^,^^13^ Patient-reported clinical scores such as single-assessment numeric evaluation (SANE), American Shoulder and Elbow Surgeons (ASES) scores, and the Western Ontario’s Rotator Cuff (WORC) scores have also been shown to improve after repair with a collagen graft.^2^, ^3^, ^4^, ^5^^,^^12^^,^^13^

Despite their promising clinical profile, collagen implants may have potential adverse outcomes, namely subacute reactive bursitis. We report two cases of reactive bursitis after rotator cuff repair with a collagen implant, capitalizing on the opportunity to evaluate graft integration and native tissue quality and rule out infectious or inflammatory reactions to the graft. These reports serve to characterize the clinical, radiographic, arthroscopic, and histologic findings after rotator cuff repair augmented with a collagen scaffold. Therein, this study provides insight into the clinical presentation, revision of surgery recommendations, and short-term outcomes.

## Case report

### Case one

The first patient is a 74-year-old, right-hand dominant male with atraumatic chronic left shoulder pain and dysfunction. The pain, which he rated as 10/10, had bothered him for years, frequently interfering with his activities of daily living. Three years earlier, the patient successfully underwent repair of a right shoulder full-thickness rotator cuff tear. His medical history included chronic obstructive pulmonary disease (COPD), formerly heavy smoking (150 pack-years), osteoarthritis, and osteoporosis.

**Figure 1:**
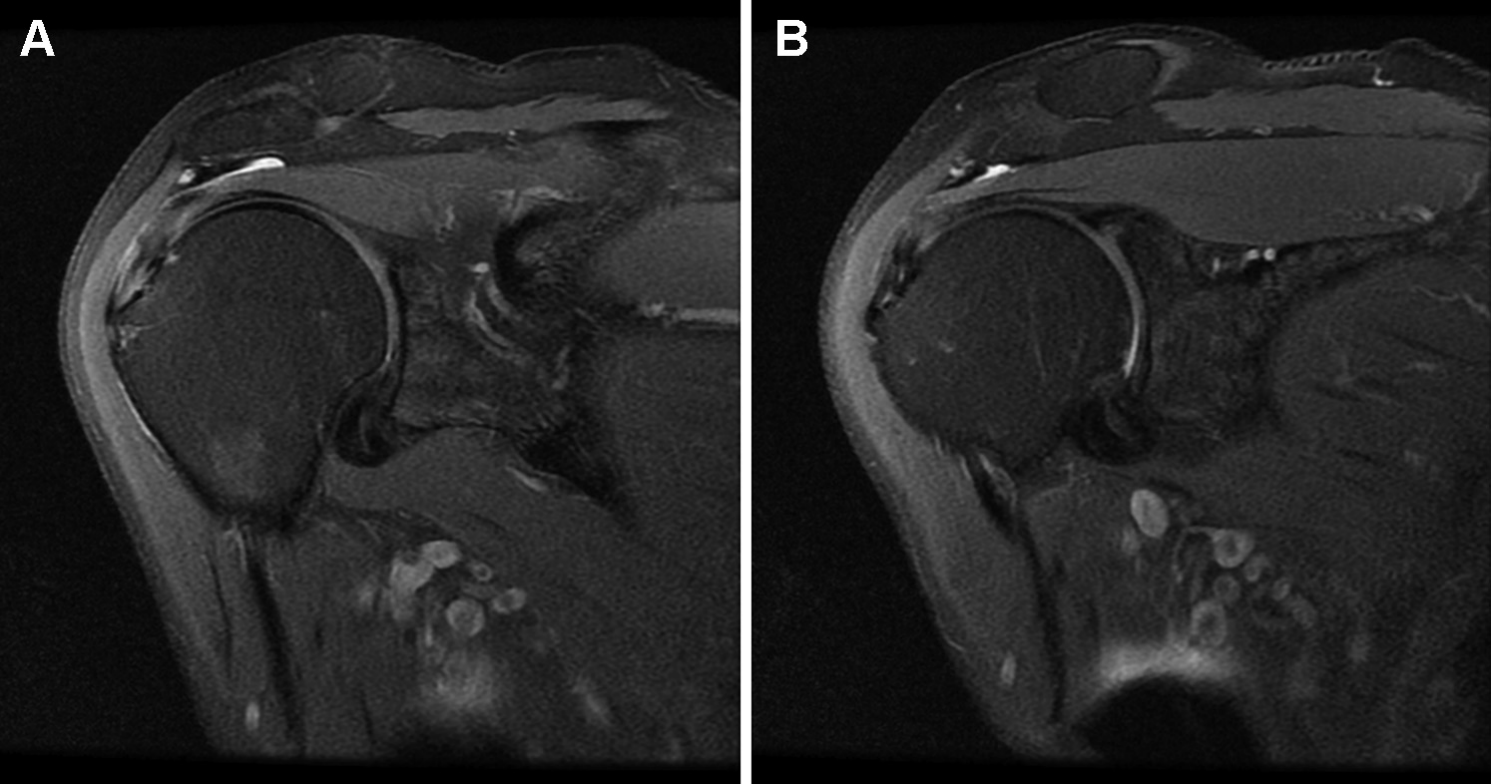
Case one - Proton dense fat-saturated coronal oblique MRI slices of the left shoulder showing high-grade tendinopathy, interstitial tearing, and thickening of supraspinatus and infraspinatus tendons. No evidence of full-thickness rotator cuff tears.

On examination, the patient exhibited diminished active abduction and forward elevation, pain with passive external rotation with no external rotation lag, tenderness to palpation over the bicipital groove, and positive impingement signs. MRI at presentation showed intrasubstance partial-thickness high-grade tears of the left supraspinatus and infraspinatus tendons and biceps tendinopathy (Fig. 1). Given that extensive conservative therapies, including several rounds of physical therapy and corticosteroid injections, did not relieve his pain, the patient was not interested in additional conservative management. The risks and benefits of surgical intervention were explained, and the patient elected to proceed with surgery.

**Figure 2:**
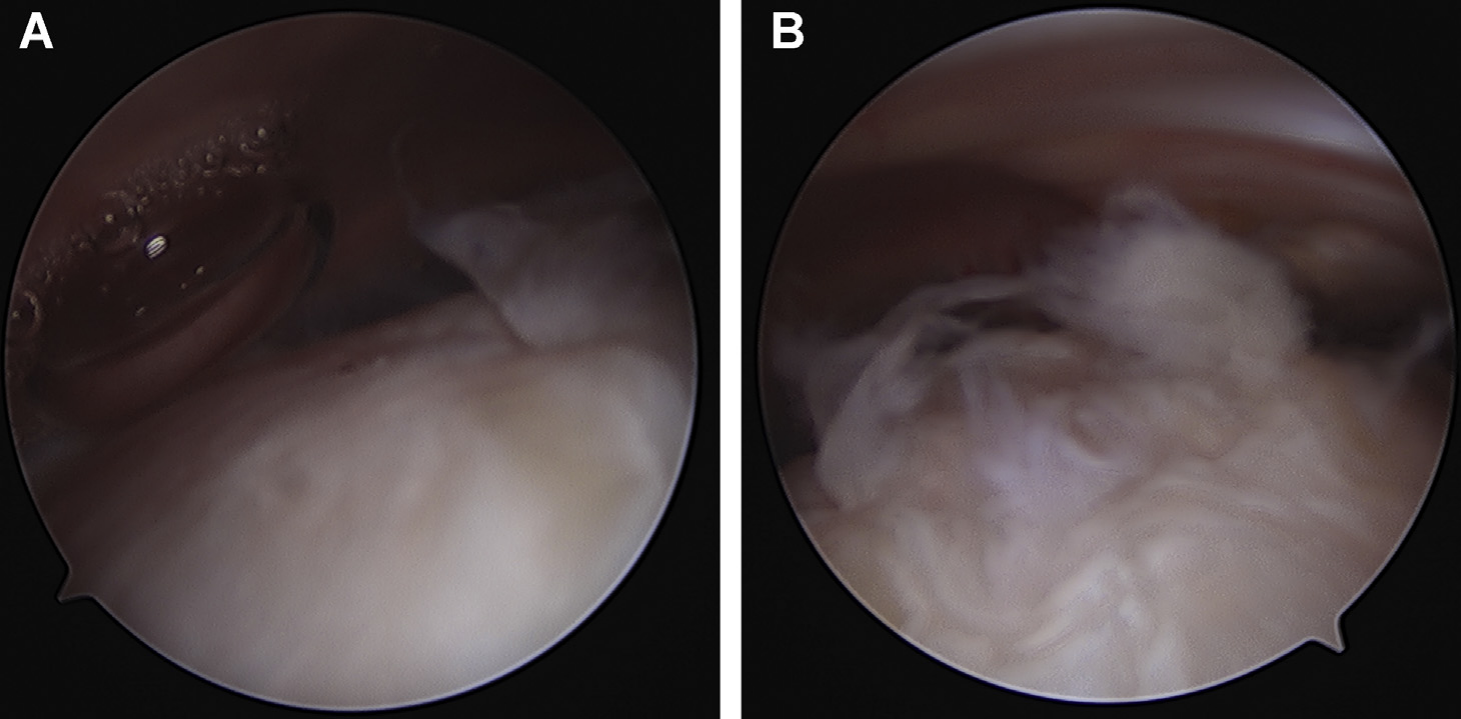
Case one intraoperative arthroscopic shoulder images revealing supraspinatus partial thickness tear.

The arthroscopic procedure occurred four months after the initial evaluation. Peripheral nerve block and general anesthesia were given without complication. Glenohumeral portals were established in the posterior soft spot and rotator interval. The intra-articular portion of the long head of the biceps tendon was noted to have significant erythema and synovitis. It was detached from its anchor at the superior glenoid labrum and retrieved via a separate open subpectoral incision and tenodesis. Subacromial arthroscopic evaluation revealed partial-thickness fraying of the bursal side of supraspinatus without a full-thickness tear (Fig. 2). Because of the extensive tendinopathy noted on MRI and partial tearing of the supraspinatus, a collagen scaffold was placed to augment the supraspinatus. The scaffold was introduced into the subacromial space through the lateral portal, deployed, and then secured on top of the existing cuff with four poly L-lactide (PLLA) sutures and two lateral bone staples.

**Figure 3:**
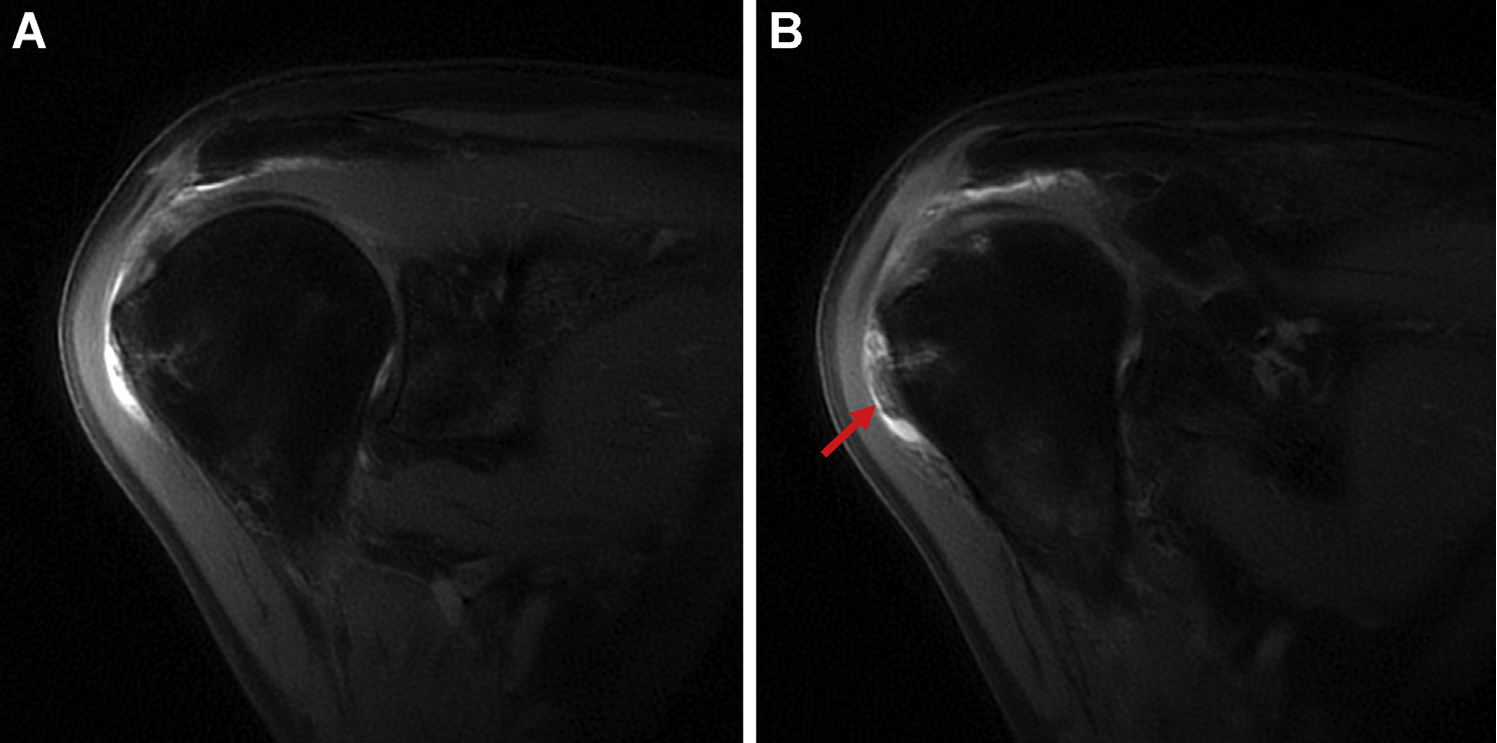
Case one follow-up - proton dense fat-saturated MRI; coronal oblique sections evidenced integration of the collagen scaffold, improvement in interstitial tearing of the rotator cuff, and a new reactive subacromial bursitis. There is also notable scar tissue in the lateral subdeltoid recess ().

For the initial two months postoperatively, the patient did well, with minimal reports of pain and improving functionality with physical therapy. However, three months postoperatively, the patient reported an acute atraumatic increase in left shoulder pain, not relieved with conservative management and rated as 10/10. An MRI taken three months postoperatively showed improvement in rotator cuff tendon thickness and decreased signal heterogeneity at the site of intrasubstance tear; however, there was subacromial and subdeltoid fluid consistent with reactive bursitis (Fig. 3). The patient failed conservative treatments, and after discussing the risks and benefits, the patient consented to an arthroscopic evaluation and débridement to resolve his symptoms and exclude an infectious etiology.

**Figure 4:**
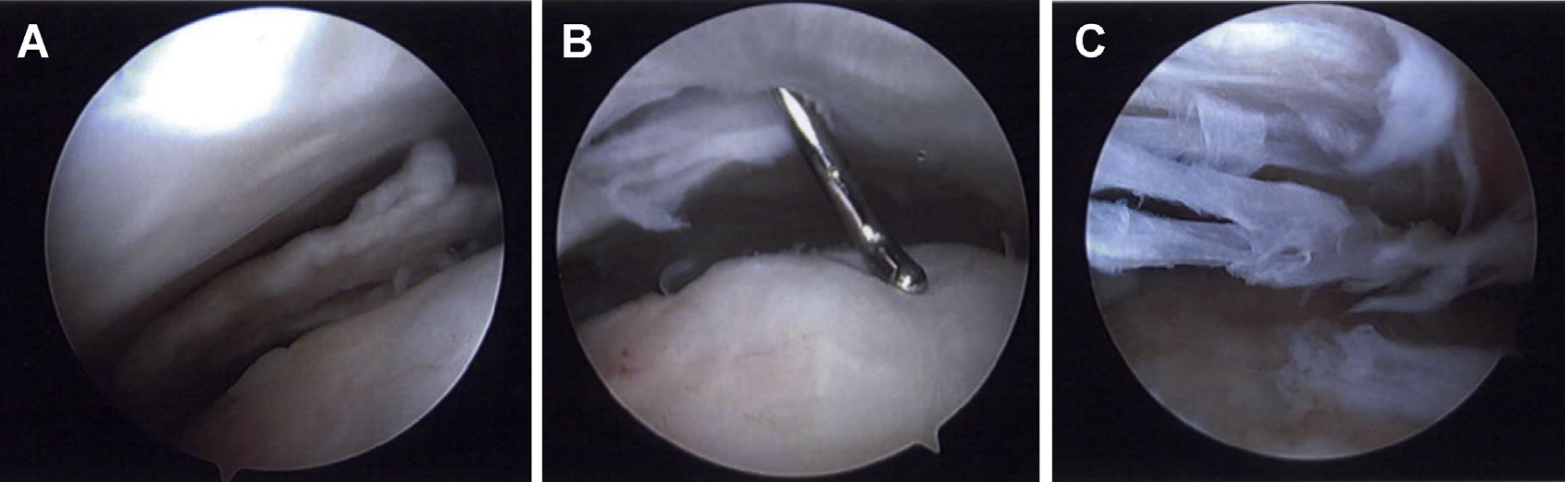
Arthroscopic images on case one revision surgery showed (**A**) the superficial portion of the bioinductive collagen scaffold had delaminated and flipped into the subdeltoid recess, corresponding to the scar tissue seen in Figure 3. (**B**) The remaining scaffold appeared to have integrated into the rotator cuff such that the rotator cuff appeared and felt normal to probing. (**C**) Significant subacromial adhesions were also noted.

**Figure 5:**
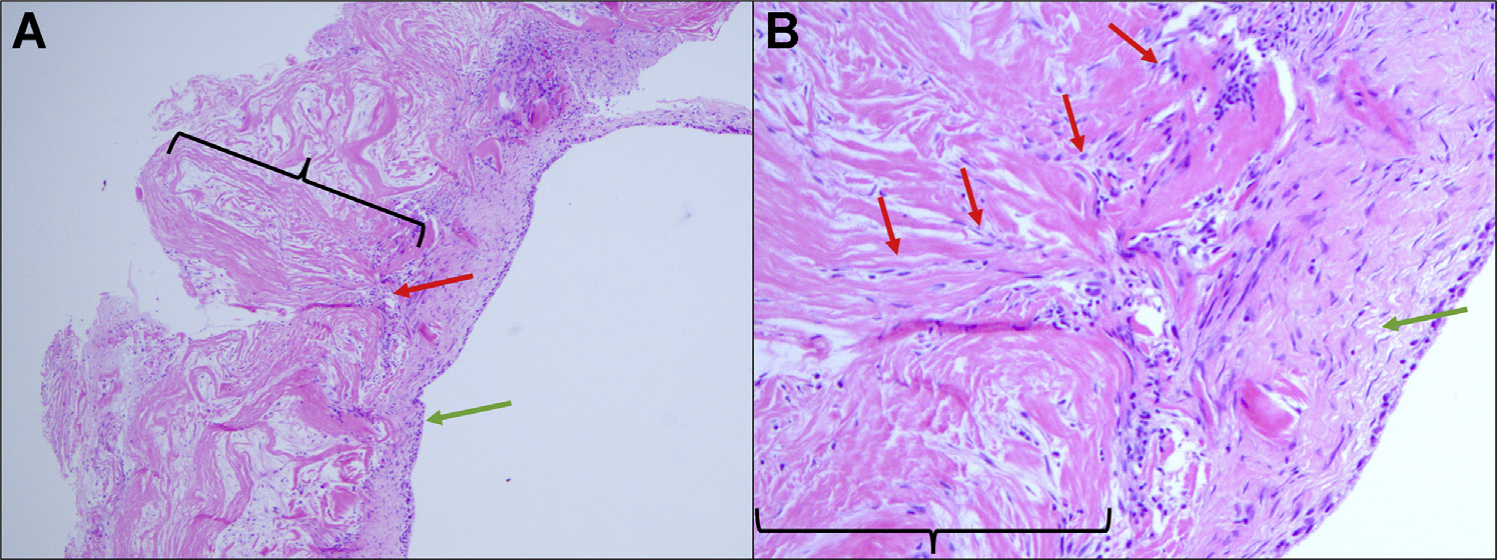
Histology images from case one revision surgery scaffold biopsy at 5X (**A**) and 10X (**B**). The biopsy was harvested at the time of the arthroscopic revision surgery and was approximately 1.05 cm^3^ in size. The specimen was fixed in 10% buffered formalin and subjected to standard automated tissue processing prior to embedding in paraffin. 5μ sections were cut and then stained with Hematoxylin and Eosin (H&E). The collagenous scaffold () was successfully captured on biopsy, as evidenced by its characteristic eosinophilic (pink) stain, acellularity, cellular paucity, and fiber morphology. Spindle-shaped, basophilic (hematoxylin/purple-stained) fibroblasts () are seen invading the collagen scaffold. Note no evidence of inflammatory cells against the scaffold. On the rightmost edge of the specimen (), there is a healthy-appearing synovial lining present, with multiple layers of fibroblast-like type B synoviocytes overlain by rounded macrophage-like type A synoviocytes. There is no evidence of pathological hyperplasia or inflammation of the synovium.

The revision surgery occurred the following month. Arthroscopic portals were established as described above, and arthroscopic evaluation showed that the glenohumeral joint was unremarkable. Subacromial evaluation, however, demonstrated significant subacromial and subdeltoid inflammation and adhesions. While the deep layer of the scaffold was well-integrated into the underlying rotator cuff, the superficial layer of the collagen scaffold had delaminated and flipped into the subdeltoid recess. Representative scar tissue in the lateral subdeltoid recess noted on MRI (Fig. 3) was similarly observed arthroscopically. The remaining scaffold, however, seemed to have integrated with the now normal appearing rotator cuff (Fig. 4). The loose, superficial scaffold material was removed from the subdeltoid recess, and the two lateral PEEK bone staples and any visible PLLA sutures were removed. A complete bursectomy was performed, and adhesions were débrided along the subacromial space, lateral, medial, and anterior borders of the acromion, and subdeltoid recess. The free-floating piece of graft, bursal tissue, staples, and sutures were sent for histologic assessment. Similarly, all samples, including the biopsied scaffold, bursa, PLLA sutures, and PEEK bone staples, were sent for microbiologic cultures and sensitivities. Anaerobic cultures were followed for three weeks and resulted in no growth. The histological evaluation confirmed fibroblast invasion into the scaffold without evidence of infection or a foreign body inflammatory reaction against the graft (Fig. 5).

The patient completed postoperative physical therapy, and at seven months after his revision surgery, he has been doing well, with his pain much improved immediately after the second arthroscopy. He experienced no further complaints.

### Case two

**Figure 6:**
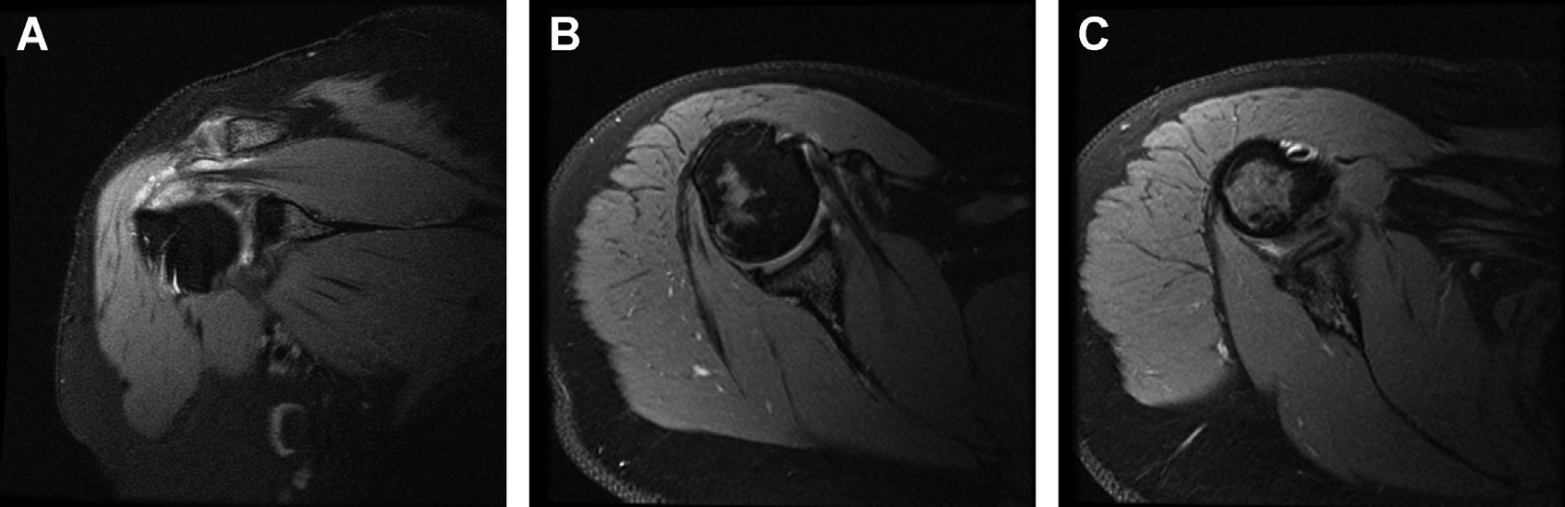
Proton dense fat saturated MRI images from case two showed partial-thickness tears of the supraspinatus, infraspinatus (**A**. coronal oblique section), and subscapularis (**B**. axial section) on a background of underlying tendinosis. (**A**) There was evidence of subacromial bursitis as well. (**C**) On the axial cut, biceps tendonitis is apparent.

**Figure 7:**
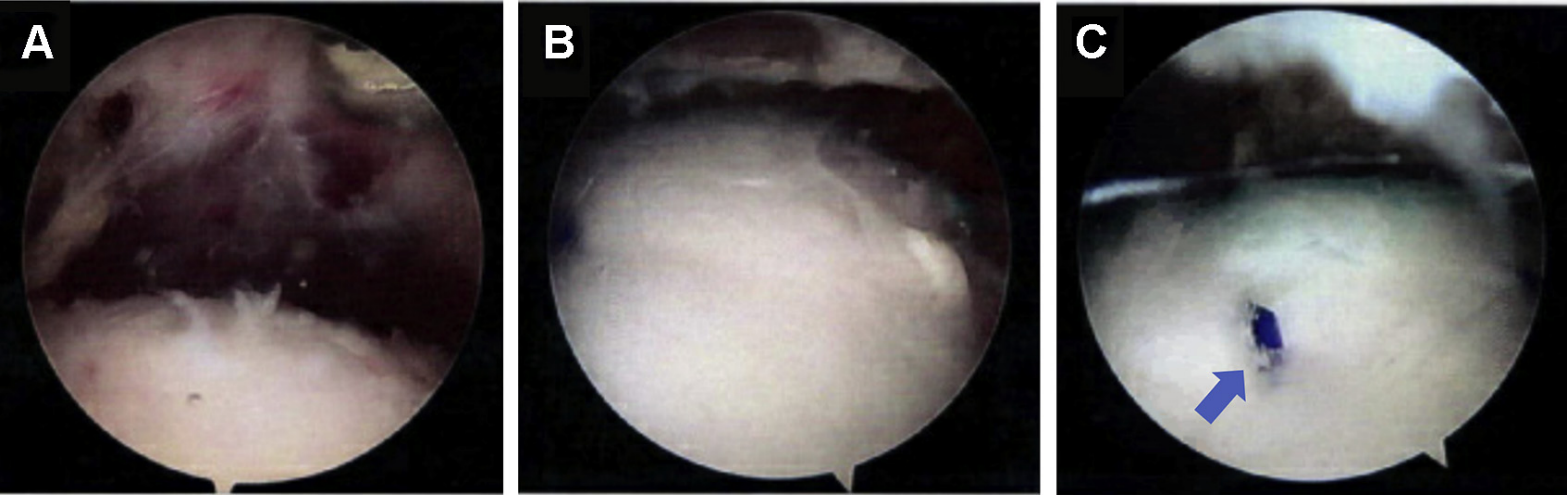
Arthroscopic images from rotator cuff augmentation for Case 2, evidencing (**A**) partial thickness fraying of posteriorsuperior cuff, with (**B**) areas of thickening. (**C**) The collagen graft was implanted with PLLA suture ().

The second patient is a 49-year-old, right-hand-dominant female with a past medical history of migraines and hysterectomy who presented to her primary care physician with two months of sharp, constant, 7/10 right shoulder pain worsened by activity, specifically reaching overhead. The pain started after the patient fell out of bed onto her right shoulder. CT demonstrated no evidence of dislocation, subluxation, or injury. On presentation to our clinic three months later, her right shoulder pain persisted. On examination, she exhibited a positive Speed’s sign, tenderness to palpation over the long head of the biceps groove and acromioclavicular joint, and positive rotator cuff impingement signs. MRI revealed partial-thickness tearing of the right supraspinatus, infraspinatus, and subscapularis superimposed on high-grade tendinosis, as well as biceps tendinitis and subacromial bursitis (Fig. 6). Given her significant symptomatology and lack of response to nonsurgical treatments, the patient wished to pursue surgical management after discussing the risks, alternatives, and benefits of surgical intervention. Two months later, the patient underwent an arthroscopic right shoulder synovectomy and débridement, distal claviculectomy, biceps tenodesis, and a rotator cuff augmentation with the same type of collagen scaffold (Fig. 7), as described above in case one.

**Figure 8:**
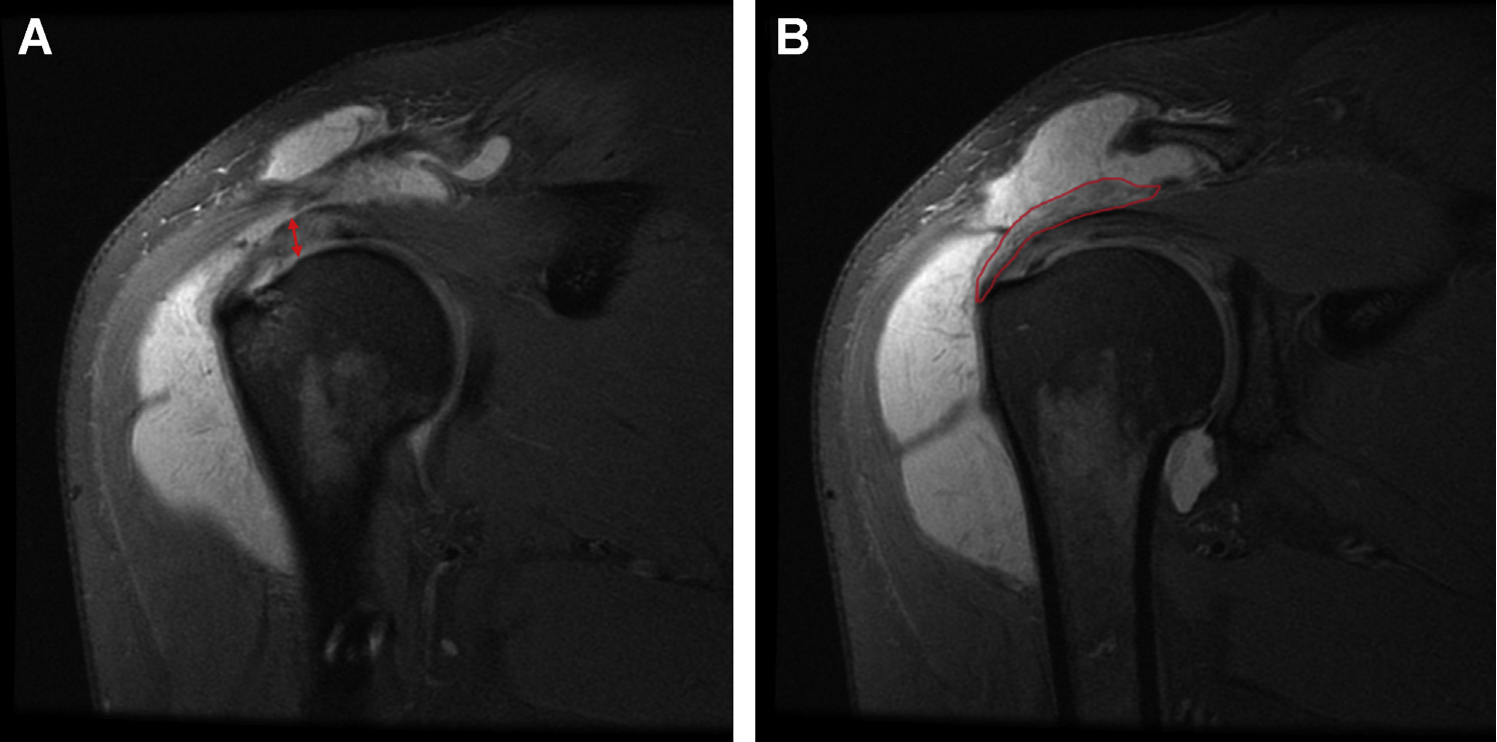
Case two follow-up proton dense fat-saturated MRI coronal sections revealing massive subacromial and subdeltoid fluid. Also noted is improvement in rotator cuff thickness compared to preoperatively (), as well as continued adhesion of the graft to the bursal side of the posterosuperior cuff ().

**Figure 9:**
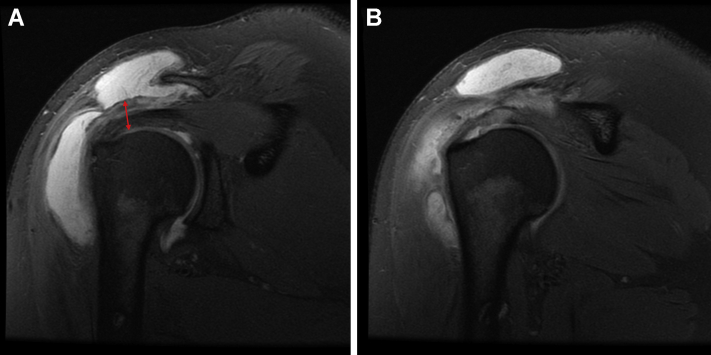
Third proton dense fat-saturated MRI for case two, after arthrocentesis and conservative management. Coronal slices demonstrate continued subacromial bursitis with evidence of progressive hypertrophy of the bursal rotator cuff tendons () and graft integration.

The patient recovered well until five months postoperatively when she injured her right shoulder while lifting her fallen mother from the floor. Her active range of motion, which had returned to full after the surgery, was diminished, and she exhibited impingement signs. A subacromial corticosteroid injection temporarily alleviated her pain. Due to the recurrence and persistence of her symptoms, she underwent a repeat MRI. This demonstrated exuberant subacromial bursitis with diffuse synovial proliferation. Compared to the preoperative MRI, her rotator cuff tendon exhibited decreased T2-weighted signal heterogeneity suggesting intrasubstance healing, albeit with some continued low-grade intrasubstance partial tear of the rotator cuff (Fig. 8). Given the amount of subacromial fluid and concern for infection, an aspiration was performed that was negative for an infectious process after holding anaerobic cultures for 3 weeks. On re-evaluation three months later, her pain persisted, unrelieved by medical management, aspiration, and corticosteroid injection. A third MRI was performed that demonstrated progressive improvement of the posterosuperior rotator cuff tendons with further decreased heterogeneity suggestive of continued healing. However, the marked bursal proliferation and subacromial bursitis persisted (Fig. 9). Treatment options, including risks and benefits, were discussed, and the patient elected for surgical management.

**Figure 10:**
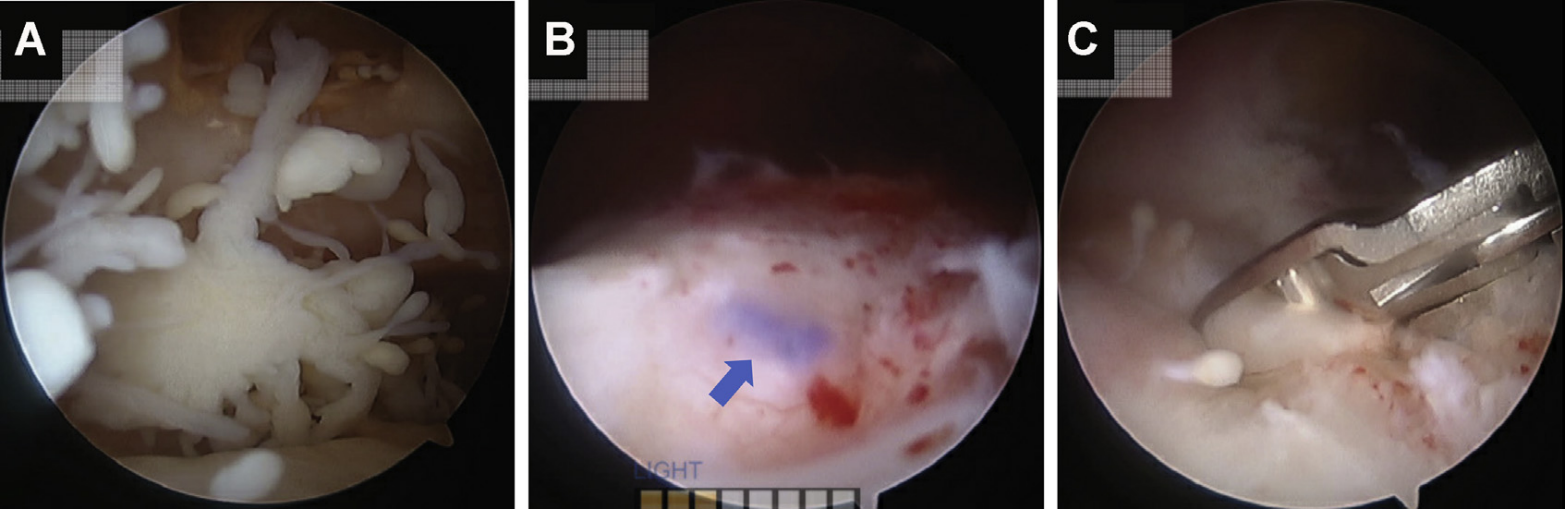
Case two revision surgery arthroscopic imaging indicated (**A**) subacromial adhesions and (**B**) inflamed bursa overlying integrated graft with PLLA suture (). (**C**) Biopsies were harvested from tissue where the graft had been implanted.

**Figure 11:**
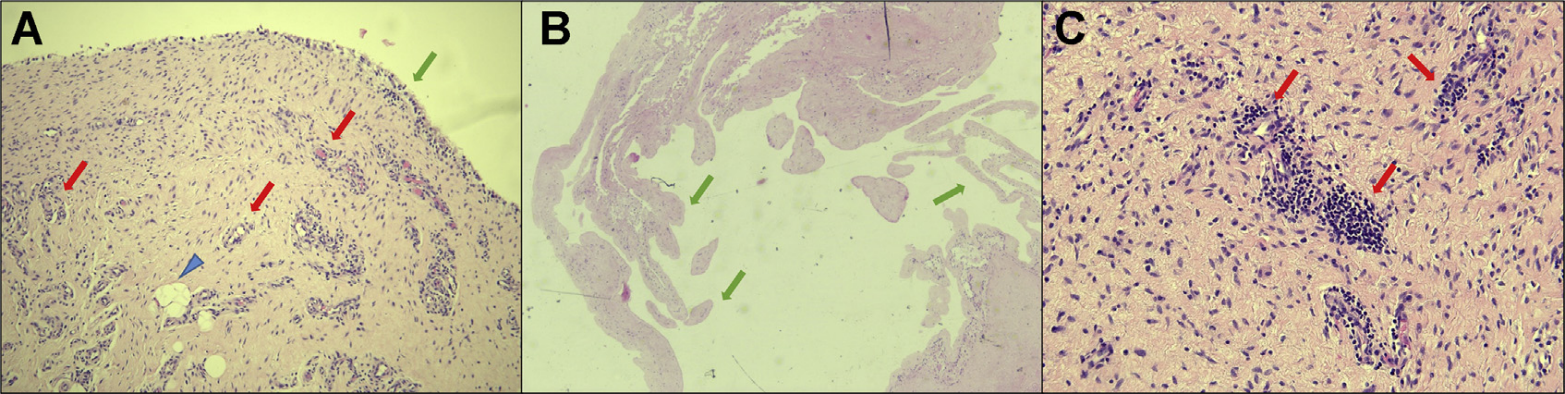
Histopathologic findings in revision surgery specimens from case two, in which hyperplastic changes were identified. Specimens for histopathological examination were collected during revision arthroscopic surgery. 10% buffered neutral formalin was used for fixation. After fixation, ethyl alcohol was used for dehydration, followed by xylene for clearing of alcohol, and paraffin was used for tissue infiltration for sectioning, using a Leica Peloris or a VIP Tissue Teks tissue processor. After the tissue was embedded in paraffin, 5 micron-thick sections were cut with a microtome and mounted on glass microscope slides. These slides were stained with hematoxylin and eosin-based protocol using an automatic stainer. (**A**) Areolar form of normal synovium with a continuous layer of lining cells (), deeper lymphovascular channels (), and occasional adipocytes (). (**B**) Fibrous form of the synovium with frond-like/papillary hyperplastic change (). (**C**) Areolar form of the synovium with focal chronic inflammation manifested by perivascular lymphocytic infiltrates (). A separate collagenous scaffold can no longer be distinguished.

Revision surgical arthroscopy occurred two months later, and intraoperative arthroscopy showed intact rotator cuff tendons, abundant subacromial bursitis and adhesions, and fraying of the coracoacromial ligament (Fig. 10). The subacromial space was débrided of adhesions, and samples along with a biopsy of the graft (Fig. 10, *C*) were obtained and sent for both microbiological culture and histopathology. As described in case one, the microbiological assessment included samples from the graft, adhesions, bursa, bone staples, and sutures sent for extended culture, sensitivities, and microscopy; all infectious workup was negative. A subacromial decompression was performed. Histology showed hyperplastic synovial tissue with focal inflammation (Fig. 11). The histology demonstrated complete integration of the graft with native tissue, as seen on arthroscopic images (Fig. 10, *C*). On the initial two-week postoperative visit, the patient was recovering well with no reports of residual pain or loss of function. Her symptoms of bursitis, swelling, and pain at rest were resolved.

## Discussion

Symptomatic rotator cuff tears requiring surgical management are common, and new management techniques and biomedical developments continue to emerge.^10^ As these tools are implemented, it remains important to assess not only outcomes but also mechanisms of action and related adverse outcomes so that we may define better surgical indications and inform patients of inherent risks. One such tool at the surgeon’s disposal is a collagen scaffold for rotator cuff augmentation, and thus far, outcomes have been promising.^2^, ^3^, ^4^^,^^9^^,^^12^^,^^13^ In the current study, we present two patients requiring revision surgery, out of a single surgeon’s cohort of over 40 patients, successfully undergoing treatment employing this implant. In the two reported cases, initially, after the index surgery, both patients showed improved range of motion and decreased pain preoperatively. Similarly, after augmentation with the collagen scaffold, both patients evidenced tendon recovery on MRI and by histopathology. Collagen scaffold augmentation is an effective way to enhance rotator cuff health by improving tendon quality and clinical outcomes, but there is the risk of a delayed-inflammatory response and partial graft delamination that should be mentioned as potential complications.

Because both case reports underwent revision surgeries, we had the unique opportunity to investigate arthroscopically and histologically how the collagen scaffold interacted with native cuff tissue. The arthroscopic findings agreed with postoperative MR imaging of decreased signal heterogeneity throughout the rotator cuff, suggesting improvement in intrasubstance structure following the addition of a collagen scaffold. In case one, a portion of the scaffold was delaminated. Our histological analysis of the intact and adhered part of the scaffold revealed that this portion of the scaffold showed histological integration with native tendon, compatible with the notably improved tendon on MR imaging. This correlates well with the findings of histologic integration reported in previous large animal^15^ and preliminary human studies.^1^ At four months after implantation, case one reveals early-stage integration with host fibroblast invasion into the graft; whereas in case two, by the time of revision surgery, about ten months after implantation, the graft seems to be fully absorbed and integrated into host tissue, corroborating well with *Arnoczky et al*’s^1^ timeline for graft resorption. Importantly, we found no evidence of foreign body inflammatory reaction to the graft itself, suggesting good biocompatibility with this scaffold. Thus, the scaffold seemed to integrate, promoting host tissue ingrowth and hypertrophy.

Both patients required revision surgeries due to reactive bursitis – perhaps a reaction to the graft-induced changes, to the absorbable components of the graft, or perhaps the joint manipulation and graft installation simply put these patients at a higher risk of developing reactive bursitis from subsequent minor trauma. Histopathologic investigation revealed focal synovial inflammation, consistent with reactive bursitis. Infectious causes were considered, given symptomatology and insertion of a foreign body; however, appropriate infectious workup proved to be negative, and both cases recovered without antimicrobial use, lending the cases to be consistent with a reactive rather than infectious bursitis. Certainly, the portion of a scaffold that was delaminated in case one may have contributed to the case’s reactive bursitis through mechanical irritation and microtrauma with the unsecured graft.

Although the focus of this study was not on reactive bursitis associated with the use of a collagen implant, our findings on this complication are consistent with other studies; one patient (of 13) in Bokor *et al*’s^3^ study developed severe bursitis 12 months status-post rotator cuff repair, and upon débridement, tissue cultures and biopsies were found to have no evidence of infection. Bushnell *et al*^5^ found that one patient (of 155) required débridement and lysis of adhesions due to inflammatory changes seen on MRI three months status-post rotator cuff repair. One patient (of 173) in McIntyre *et al*’s^9^ study experienced postoperative recurrent effusions and bursitis requiring débridement and synovectomy. Importantly, we found that even in the setting of our cases of bursitis, graft integrity of incorporated portions under histologic evaluation was maintained. Further research should be done to better elicit the possible association between collagen graft augmentation and reactive bursitis.

## Conclusion

Rotator cuff repair or augmentation with a collagen scaffold augmentation enhances rotator cuff tendon thickness, maturity, and function. The histologic evaluation confirms that the collagen implant integrates with native tissue and does not appear to cause a foreign body reaction, even in the setting of reactive bursitis. Patients undergoing collagen scaffold augmentation should be warned of the possibility of reactive bursitis. Cases of reactive bursitis may need to be managed with surgical débridement to treat symptoms and structural failure and rule out infection with good results. Despite this uncommon complication, our histologic and MRI findings support the continued use of this implant for severe rotator cuff tendinosis and partial thickness tearing.

## Disclaimers:

Funding: No funding was disclosed by the authors.

Conflicts of interest: The authors, their immediate families, and any research foundation with which they are affiliated have not received any financial payments or other benefits from any commercial entity related to the subject of this article.
